# [Retracted] Long non‑coding RNA BCYRN1 promotes glycolysis and tumor progression by regulating the miR‑149/PKM2 axis in non‑small‑cell lung cancer

**DOI:** 10.3892/mmr.2024.13380

**Published:** 2024-11-04

**Authors:** Ning Lang, Chunyang Wang, Jiangyang Zhao, Feng Shi, Tong Wu, Hongyan Cao

Mol Med Rep 21: 1509–1516, 2020; DOI: 10.3892/mmr.2020.10944

Following the publication of this paper, it was drawn to the Editors’ attention by a concerned reader that the Transwell cell invasion data shown in Fig. 4D were strikingly similar to data appearing in different form in other articles written by different authors at different research institutes that had already been published elsewhere prior to the submission of this paper to *Molecular Medicine Reports* (one of which has been retracted). In view of the fact that certain of the abovementioned data had already apparently been published previously, the Editor of *Molecular Medicine Reports* has decided that this paper should be retracted from the Journal. The authors were asked for an explanation to account for these concerns, but the Editorial Office did not receive a reply. The Editor apologizes to the readership for any inconvenience caused.

